# High Protein Diet and Huntington's Disease

**DOI:** 10.1371/journal.pone.0127654

**Published:** 2015-05-19

**Authors:** Chiung-Mei Chen, Yow-Sien Lin, Yih-Ru Wu, Pei Chen, Fuu-Jen Tsai, Chueh-Lien Yang, Ya-Tzu Tsao, Wen Chang, I-Shan Hsieh, Yijuang Chern, Bing-Wen Soong

**Affiliations:** 1 Department of Neurology, Chang Gung Memorial Hospital, Linkou Medical Center and College of Medicine, Chang-Gung University, Taoyuan, Taiwan; 2 Division of Neuroscience, Institute of Biomedical Sciences, Academia Sinica, Taipei, Taiwan; 3 Department of Pediatrics, Medical Research and Medical Genetics, China Medical University Hospital, Taichung, 404, Taiwan; 4 Department of Dietetics and Nutrition, Taipei Veterans General Hospital, Taipei, Taiwan; 5 Department of Nutrition, Chang Gung Memorial Hospital, Linkou Medical Center and College of Medicine, Chang-Gung University, Taoyuan, Taiwan; 6 Department of Neurology, Yang-Ming University School of Medicine and Neurological Institute, Taipei Veterans General Hospital, Taipei, Taiwan; University of Pennsylvania Perelman School of Medicine, UNITED STATES

## Abstract

Huntington’s disease (HD) is a neurodegenerative disorder caused by the *huntingtin* (*HTT*) gene with expanded CAG repeats. In addition to the apparent brain abnormalities, impairments also occur in peripheral tissues. We previously reported that mutant Huntingtin (mHTT) exists in the liver and causes urea cycle deficiency. A low protein diet (17%) restores urea cycle activity and ameliorates symptoms in HD model mice. It remains unknown whether the dietary protein content should be monitored closely in HD patients because the normal protein consumption is lower in humans (~15% of total calories) than in mice (~22%). We assessed whether dietary protein content affects the urea cycle in HD patients. Thirty HD patients were hospitalized and received a standard protein diet (13.7% protein) for 5 days, followed by a high protein diet (HPD, 26.3% protein) for another 5 days. Urea cycle deficiency was monitored by the blood levels of citrulline and ammonia. HD progression was determined by the Unified Huntington’s Disease Rating Scale (UHDRS). The HPD increased blood citrulline concentration from 15.19 μmol/l to 16.30 μmol/l (*p* = 0.0378) in HD patients but did not change blood ammonia concentration. A 2-year pilot study of 14 HD patients found no significant correlation between blood citrulline concentration and HD progression. Our results indicated a short period of the HPD did not markedly compromise urea cycle function. Blood citrulline concentration is not a reliable biomarker of HD progression.

## Introduction

Huntington’s disease (HD) is an inherited neurodegenerative disorder caused by the expansion of CAG repeats in exon 1 of the *huntingtin* (*HTT*) gene. The clinical features of HD include uncontrollable motor movements, psychiatric abnormalities, dementia, and weight loss. This devastating disease preferentially affects the cerebral cortex and striatum in the central nervous system [1]. Abnormalities in the peripheral tissues, including the cardiovascular system, skeletal muscles, blood cells, and cochlea, have also been reported [2–6]. Whether dysregulated peripheral functions can serve as reliable biomarkers of the progression of HD has been discussed [7].

Consistent with previous studies that showed the existence of mutant HTT (mHTT) in the liver [8–10], we and others have reported that aggregates of mHTT localize in the liver and that the expression of mHTT suppresses the expression of two key enzymes (argininosuccinic acid synthetase and argininosuccinase acid lyase) of the urea cycle by disrupting the transcriptional activity of C/EBP [11–13]. The resultant elevated blood citrulline level was detected in two HD model mice (R6/2 and Hdh^Q150^) and in patients with HD. Most importantly, dietary protein restriction normalized the blood ammonia and citrulline levels in HD model mice and was associated with less aggregation of mHTT, improved rotarod performance, and a higher level of striatal brain-derived neurotrophic factor. A low protein diet is thus an effective means of slowing the disease progression in HD model mice.

One of the major symptoms of urea cycle deficiency is an elevated blood ammonia level. Ammonia is a byproduct of protein metabolism and is a well-known neurotoxin. Hyperammonemia usually occurs in infants with a urea cycle disorder and in adults with liver failure [14,15]. Low protein diets have been used to treat hyperammonemia to reduce the ammonia level in the circulation [16,17], however, the efficacy of this approach to treat hepatic encephalopathy in cirrhosis has been challenged [18].

Dietary therapy is of great interest for HD patients because body weight loss is associated with disease progression in HD [19–21]. Marder and colleagues reported recently that higher caloric intake and higher dairy consumption are marginally associated with earlier clinical onset of HD [22], highlighting the importance of evaluating energy expenditure and specific dietary components in presymptomatic gene carriers and in patients showing symptoms of HD. For example, a previous study of 51 Dutch families affected by HD reported that a high intake of milk and milk products was associated with early onset of HD [23]. Although the biological relevance of the above finding is unclear, it suggests that certain nutritional elements can affect the pathogenesis of HD. Although we have reported the beneficial effects of two different low protein diets (17% of total calories) in two HD model mice [13], it is unclear whether this finding can be translated to humans with HD because the normal human protein content is lower than that of mice (15% and 22% of total calories, respectively) [13,24]. In the present study, we assessed the potential impact of a short period of high dietary protein content on patients with HD (6 days, 26.3% of total calories) and evaluated whether the elevated blood citrulline level caused by impaired urea cycle function can serve as a reliable biomarker of HD.

## Materials and Methods

### Participants

Genetically confirmed HD patients were recruited from Chang Gung Memorial Hospital (CGMH) and Taipei Veterans General Hospital (TVGH) for a 12-day hospitalization in either of the hospitals. Patients with prior dietary supplementation (including creatine and Q10) were asked to discontinue the intake of supplements for at least 1 month before enrolling in this study. Patients with a prior history of liver or renal dysfunction, or pregnancy were excluded. The protocol was approved by the Institutional Review Boards at Academia Sinica, CGMH, and TVGH. Written informed consent was obtained before any study-related procedures.

### Diets

A standard protein diet (SPD, 13.7% of total calories) and a high protein diet (HPD, 26.3% of total calories) were designed by the Departments of Food and Nutrition at CGMH and TVGH based on the body weight of each individual HD patient (35 kcal/kg) on the day of enrollment and were prepared by the corresponding hospital kitchen. The SPD was given to patients from day 2 to day 6 (Stage I), and the HPD was given from day 7 to day 11 (Stage II).

### Medical examinations

The Unified Huntington’s Disease Rating Scale (UHDRS) and Mini-Mental State Examination (MMSE) were used to evaluate the patients according to the instructions of the Huntington Study Group [25]. These tests were conducted in CGMH or TVGH, as relevant for each patient. Three major features including motor function, independence, and functional capacity were scored. Fasting blood samples were collected on the morning of the indicated date and were analyzed at CGMH or TVGH to assess renal function, liver function, and ammonia levels.

### Blood citrulline level

Intravenous whole blood samples (25 μl) were spotted onto filter papers (Schleicher and Schuell No. 903), which were allowed to air dry and were stored at room temperature. Citrulline concentration was measured using a tandem mass spectrometer (Quattro Micro, Waters Corporation, Milford, MA, USA) as described previously [13,26].

### Statistical analysis

The data were analyzed using the paired Student’s *t* test. The Pearson correlation coefficient was used to identify correlations between the UHDRS score and citrulline concentration. Significance was set at *p* 0.05. Unless stated otherwise, all data are shown as mean ± SD.

## Results

### Limited effect of the 5-day HPD on urea cycle function in patients with HD

**Fig 1:**
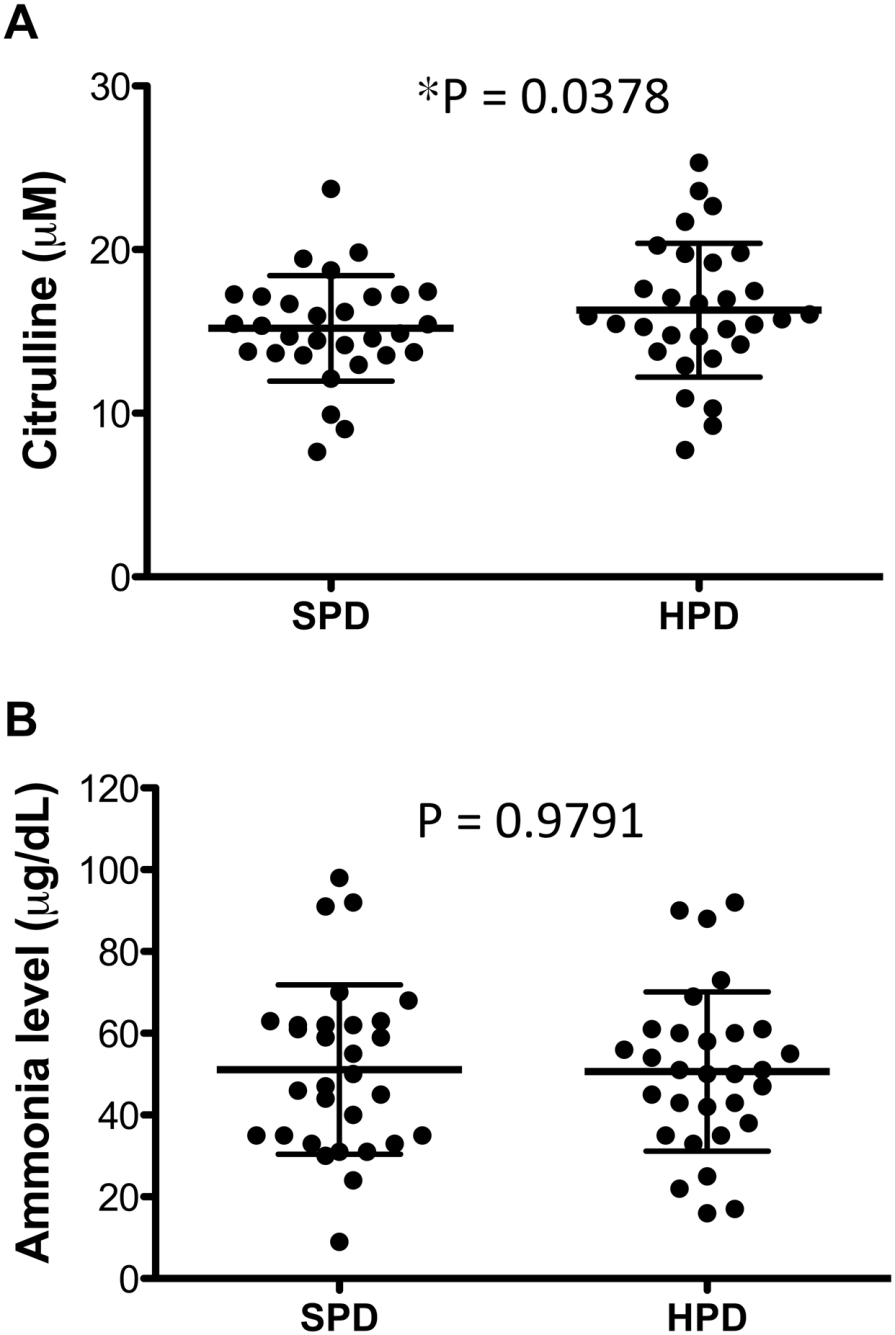
Influence of dietary protein content on the urea cycle in Huntington’s disease (HD) patients. Blood citrulline (A) and ammonia (B) levels in HD patients given the standard protein diet (13.7%) and high protein diet (26.3%). The data for the 23 non-HD subjects (Con) were taken from a previous report [13]. All HD patient data were plotted. Data are presented as mean ± standard deviation (SD) and were analyzed by paired *t* test. *p < 0.05.

Thirty patients were recruited for a 12-day hospitalization in CGMH or TVGH. Blood samples were collected on day 2. The SPD containing 13.7% protein was given to the enrolled HD patients (Table 1). On day 6 (i.e., after 5 days of the SPD), arterial blood samples were collected to measure the blood ammonia level and venous blood samples were collected to measure the blood citrulline level. Because the liver ultrasound images and the blood tests for liver function of all HD patients were normal, the enrolled HD patients were given HPD (26.3% protein) for another 5 days (from day 7 to day 11). Patients were monitored closely by a dedicated nurse in the corresponding hospital for the proper intake of daily diet and any potential adverse effect. On day 12 (i.e., after 5 days of the HPD), arterial ammonia levels and venous citrulline levels were measured. As shown in Fig 1, the 5-day HPD slightly increased the venous blood citrulline level (15.19 μmol/l on day 6 and 16.30 μmol/l on day 12; p = 0.0378) (Fig 1A). Arterial blood ammonia level was not affected by the HPD (51.10 μg/dl and 50.67 μg/dl on days 6 and 12, respectively) (Fig 1B). The normal range of blood ammonia is < 65 μg/dl (upper normal limit calculated as reported earlier [27]). Of the 30 enrolled patients with HD, only five patients had a blood ammonia level > 65 μg/dl (Fig 1B). Most importantly, the HPD had no consistent effect on the blood ammonia level (Fig 1B).

**Table 1:** Clinical characteristics of Huntington’s disease (HD) patients.

Characteristic	Non-HD controls ^a^	HD patients
**Mean age (years)**	42.26 ± 2.6	44.77 ± 11.4
**Gender**	12 men and 11 women	19 men and 11 women
**Mean onset age (years)**		37.43 ± 11.3
**Disease duration (years)**		7.34 ± 7.2
**CAG repeats**		46.47 ± 5.8

Data are presented as the mean ± SD.

^a^ The data for the 23 non-HD subjects were taken from a previous report [13].

### No correlation between the blood citrulline concentration and HD progression

**Fig 2:**
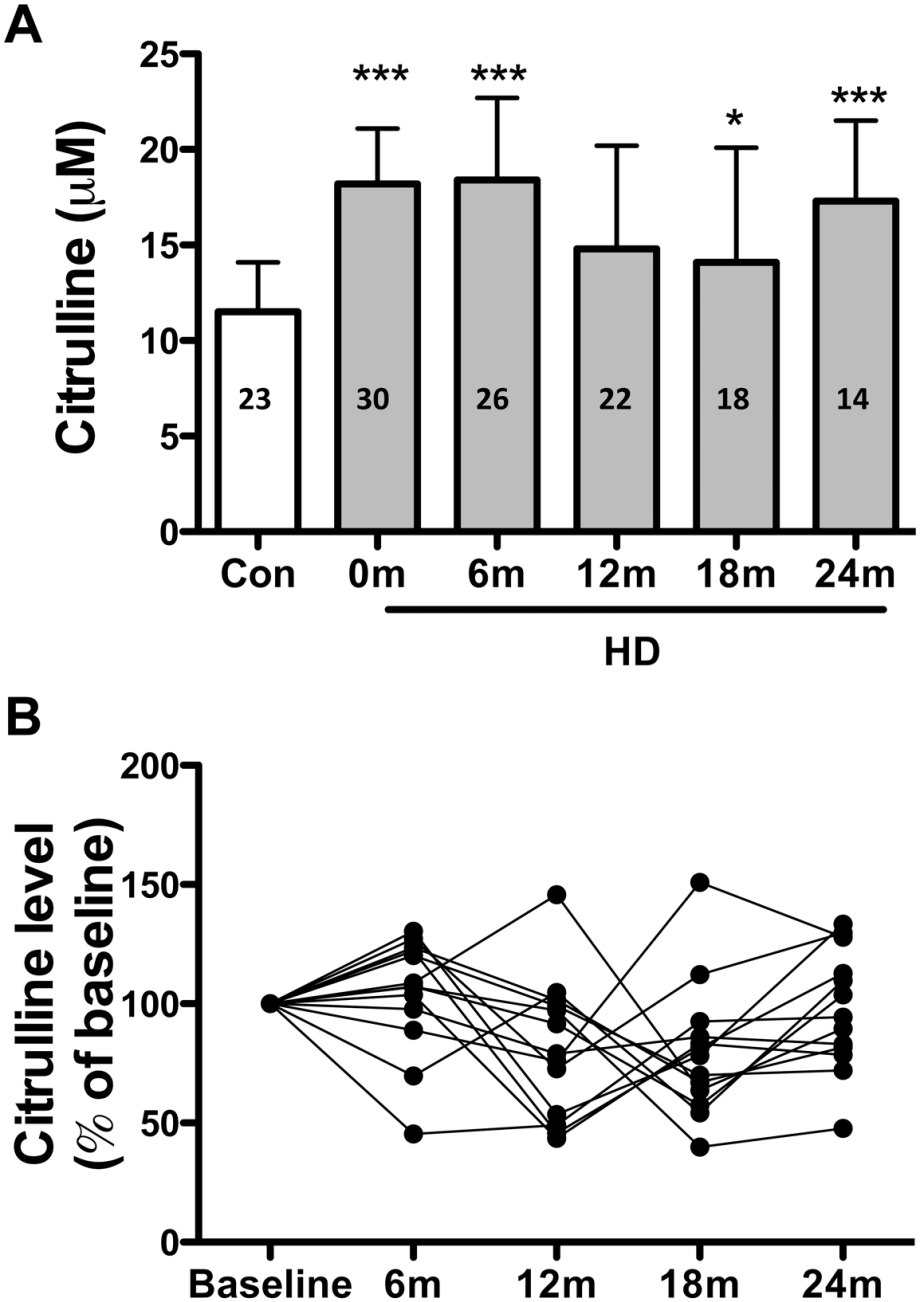
Blood citrulline concentration during follow-up of Huntington’s disease (HD) patients. Blood citrulline concentration was measured every 6 months and was compared at each time point with the concentration in non-HD controls (dotted line). The N value in the bar represents the number of collected data at each point (A). All data were normalized to the baseline value and show the progression pattern (B). Data are presented as mean ± standard deviation, *p < 0.05, ***p < 0.001 by *t* test.

Increased blood citrulline and ammonia levels are hallmarks of urea cycle deficiency. We have reported previously that the blood citrulline levels of mice and humans with HD are higher than in non-HD littermate controls and non-HD subjects, respectively [13]. To evaluate whether blood citrulline concentration can serve as a biomarker of the progression of HD, we conducted a pilot study to monitor the blood citrulline levels and HD disease progression over a 2-year period. Venous blood samples were collected every 6 months at the clinic where each patient’s UHDRS and MMSE were assessed. Although the blood citrulline concentration was higher in HD patients than in non-HD controls, as reported earlier [13] (Fig 2), the blood citrulline level did not change significantly in the HD patients during the 2-year follow-up (Fig 2A). By contrast, significantly higher motor score and lower functional capacity were observed in the HD patients at both 18 and 24 months (Table 2). Because of disease progression, only 14 of the initial 30 HD patients completed the 2-year study. In these patients, the blood citrulline concentration did not correlate significantly with the UHDRS score (S1 Fig). We also investigate the association between the disease duration and the blood citrulline level. To our surprise, the blood citrulline level negatively correlated with the disease duration of HD patients (S2 Fig).

**Table 2:** UHDRS and blood citrulline concentrations in HD patients.

Characteristic	Non-HD Subjects	HD patients				
		Baseline	6-month	12-month	18-month	24-month
**N**	23	14	14	14	14	14
**Citrulline (**μ**M)**	11.5 ± 2.6^a^	18.2 ± 2.9	18.4 ± 4.3	14.8 ± 5.4	14.1 ± 6.0^#^	17.3 ± 4.2
**UHDRS Motor score**		29.8 ± 20.5	29.4 ± 15.1	32.2 ± 13.0	39.2 ± 14.7*	45.7 ± 16.2***
**Functional capacity**		8.3 ± 4.7	7.8 ± 4.1	7.3 ± 3.6	6.4 ± 3.6***	5.6 ± 3.5***
**Independence score**		75.7 ± 25.0	77.9 ± 21.6	77.9 ± 18.9	72.1± 20.5	70.0 ± 18.8

Data are presented as mean ± SD.

^a^ The blood citrulline level of the 23 non-HD subjects was taken from a previous report [13].

*p < 0.05,

**p < 0.01,

***p < 0.001 compared with the baseline (initial enrollment of patients);

^#^p < 0.05, comparison between 6-month and 18-month; one-way repeated measures analysis of variance, followed by a Bonferroni post hoc multiple-comparisons test.

## Discussion

Energy deficit has been implicated in the pathogenesis of HD [28–31]. Dietary intervention to provide proper nutrition to HD patients has been discussed and investigated [13,21–23,32]. We have reported previously that a low protein diet (17% of total calories) was beneficial and ameliorated several major symptoms in a HD model mouse [13]. In the present study, we set out to determine whether the dietary protein intake of HD patients needs specific attention. Given that the standard protein content is lower in humans than in mice (15% and 22% of total calories, respectively) and that a well-monitored HPD can be beneficial at least in normal subjects [33], we increased the dietary protein content from 13.7% to 26.3% of total calories through the HPD and monitored the effects on urea cycle function in HD patients. We did not choose to evaluate LPD because the standard protein diet (13.7% protein), which we used for HD patients in the present study, was already much lower than the LPD (17% protein) for HD mice. In addition, to evaluate the beneficial effect of LPD on HD patients (< 13.7%), these patients need to be on LPD for at least 6 months. The safety and welfare of HD patients on a chronic LPD was a concern. Our goal in the present study was to determine whether a short period of high dietary protein content on patients with HD (6 days, 26.3% of total calories) would provoke the functional deficiency of urea cycle. The limited effect of the short-term HPD on blood citrulline (Fig 1A) and ammonia (Fig 1B) concentrations suggested that, although the urea cycle of HD patients is impaired compared with non-HD subjects, a temporary increase in dietary protein content does not markedly compromise the function of the urea cycle in HD patients. Whether chronic HPD would affect urea cycle function or disease progression in HD patients remains to be investigated.

The 2-year follow-up study of 14 patients with HD (Fig 2, Table 2) suggested that elevated blood citrulline concentration does not appear to be a reliable biomarker of HD progression. Consistent with our previous findings [13], the blood citrulline levels were significantly higher in the HD patients during this 2-year follow-up than the values reported for non-HD subjects (controls) (Fig 2A; [13]). However, no significant changes were found in blood citrulline concentration, although alterations in motor function and functional capacity were evident at 18 and 24 months (Fig 2, Table 2). In addition to serving as a surrogate endpoint marker of the urea cycle, the blood citrulline level also reflects the absorptive function of the intestine [34,35]. Food intake and absorption may also contribute to the blood citrulline level [36]. Unlike other inherited urea cycle disorders [16,37–40], the increase in blood citrulline level in HD patients is moderate [13] (Fig 2) and is more likely to be affected by the contributions of other organs such as the intestine. The surprising inverse relationship between blood citrulline concentration and disease duration (S2 Fig) also implies that the nutritional status of HD patients, especially in the end stage, might influence the blood citrulline level.

In summary, the results of this pilot study suggest that normal dietary protein content may not be high enough to impair urea cycle function in HD patients. The blood citrulline concentration does not seem to be a sensitive and reliable biomarker of HD progression.

## Supporting Information

S1 Fig Correlation between citrulline level and UHDRS. Citrulline levels of all HD patients within two years follow up data were correlated with motor score (A, *P* = 0.0985, *r* = -0.1584), independence scale (B, *P* = 0.2144, *r* = 0.1193), and functional capacity (C, *P* = 0.5010, *r* = 0.06484).
(TIF)

S2 Fig Citrulline level negative correlate with disease duration of HD. Citrulline levels of 12-month follow up data (n = 22) were correlated with disease duration (*P* = 0.0221, *r* = -0.4745), *p < 0.05, Pearson’s correlation.
(TIF)
