# Supplementary figures and images for: High Protein Diet and Huntington's Disease
Source: PLoS One. 2015 May 19;10(5):e0127654. doi: 10.1371/journal.pone.0127654 (PMC4437787; doi:10.1371/journal.pone.0127654)

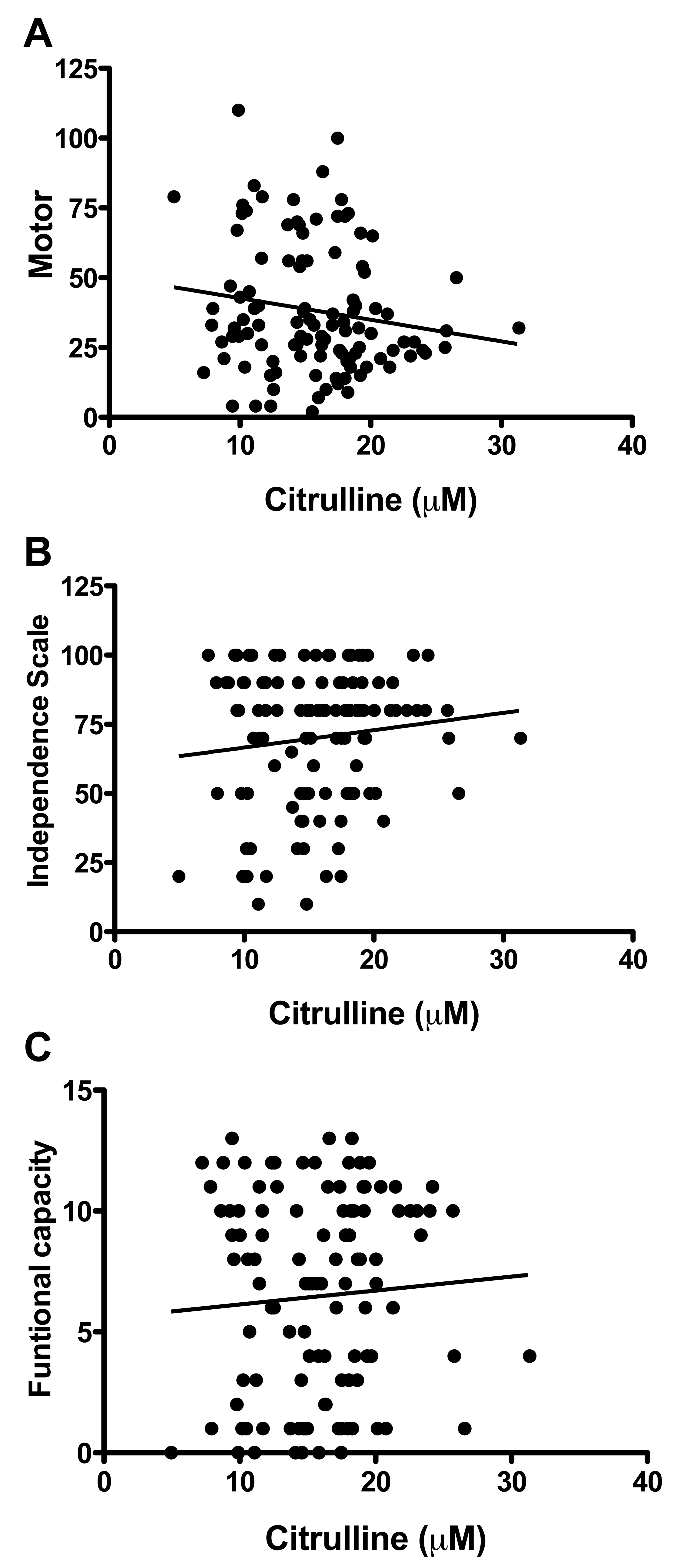

Supplement: S1 Fig — Citrulline levels of all HD patients within two years follow up data were correlated with motor score (A, P = 0.0985, r = -0.1584), independence scale (B, P = 0.2144, r = 0.1193), and functional capacity (C, P = 0.5010, r = 0.06484). (TIF) [file pone.0127654.s001.tif]

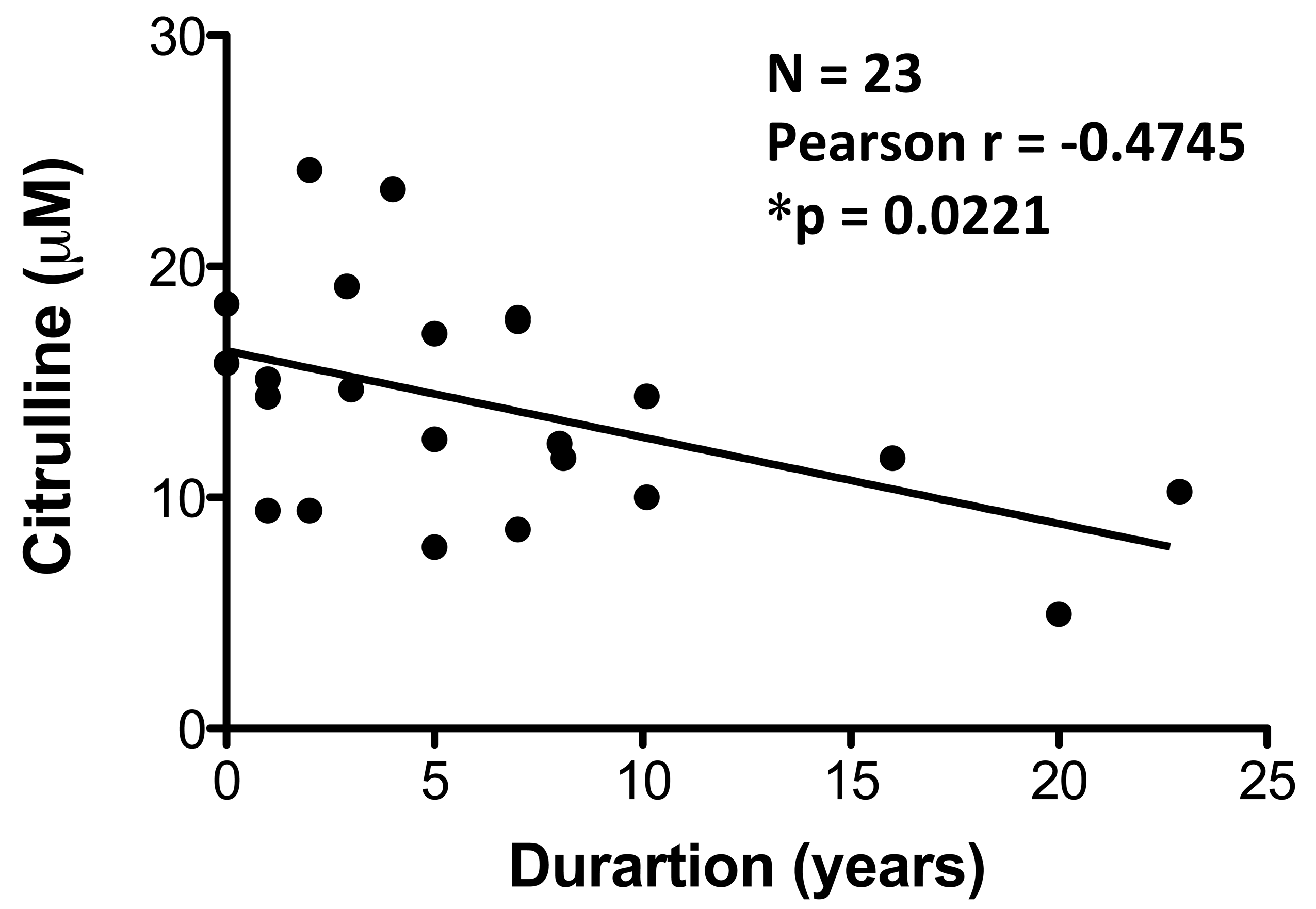

Supplement: S2 Fig — Citrulline levels of 12-month follow up data (n = 22) were correlated with disease duration (P = 0.0221, r = -0.4745), *p < 0.05, Pearson’s correlation. (TIF) [file pone.0127654.s002.tif]
